# Clinical follow-up on a cohort of patients with deficiency of adenosine deaminase 2 (DADA2)

**DOI:** 10.1186/1546-0096-13-S1-O21

**Published:** 2015-09-28

**Authors:** K Barron, A Ombrello, D Stone, P Hoffmann, I Aksentijevich, Q Zhou, A Jones, D Kastner

**Affiliations:** 1 NIAID, NIH, Bethesda, USA; 2 NHGRI, NIH, Bethesda, USA

## Introduction

We previously reported a syndrome of intermittent fevers, early-onset lacunar strokes, livedoid rash, hepatosplenomegaly, immune deficiency, and systemic vasculopathy, associated with loss-of-function mutations in *CECR1*. An additional report by others expanded the clinical spectrum to include patients with cutaneous polyarteritis nodosa.

## Objectives

We now present clinical follow-up on our reported 5 patients and an additional 9 patients.

## Patients and methods

We evaluated the 14 patients at the National Institutes of Health. All patients were enrolled in an IRB approved study. We performed whole-exome sequencing in the initial 3 patients and their unaffected parents and candidate-gene sequencing in the other 11 patients. Clinical information and radiographic and laboratory testing were obtained at each visit.

## Results

All patients had 2 mutations in *CECR1*. 11/14 patients reported recurrent fevers.

10/14 patients had at least one stroke, with 8/10 before the age of 5 years. Magnetic resonance imaging showed evidence of acute or chronic small subcortical infarcts involving the deep-brain nuclei and the brain stem, consistent with small-vessel occlusions (lacunar strokes). Three patients had additional hemorrhagic strokes. In 10/10 patients, magnetic angiography showed no evidence of cerebral vasculitis.

All 14 patients demonstrated livedo racemosa. Erythematous papules or nodules were seen in 11 of these patients.

Hepato- and/or splenomegaly was observed in 10/14 with 3 patients demonstrating portal hypertension. One patient developed a perforated small bowel requiring resection.

Hypertension was noted in 2 patients. Prolonged QT was reported in 3 patients.

12/14 demonstrated hematologic abnormalities including anemia, leukopenia, and/or thrombocytopenia. Elevation of acute phase reactants was reported in 13/14 patients.

Low serum iron was noted in 8/10 patients tested.

10/13 presented with hypogammaglobulinemia, however, this may reflect prior treatment with cyclophosphamide in 3 patients.

Most patients had received a number of medications over the course of their disease. It was our practice to discontinue aspirin and/or anticoagulation in all of our DADA2 patients. We observed striking improvement in CRP, ESR, CBC, and serum iron in 10/12 patients receiving anti-TNF agents.

## Conclusion

We have expanded the clinical picture of our cohort of patients with DADA2 to include multiple strokes, livedo racemosa, cutaneous PAN, portal hypertension, hematologic abnormalities, vascular pathology and mild immunodeficiency. In addition, we have demonstrated both clinical and laboratory improvement following treatment with anti-TNF agents.

